# An 18-year Experience with an Innovative Geriatrics Training Model: Implications for the Workforce

**DOI:** 10.1093/geroni/igab046.2808

**Published:** 2021-12-17

**Authors:** Cara O'Brien, Evan Henricks, Angela Beckert, Kathryn Denson, Edmund Duthie

**Affiliations:** 1 Medical College of Wisconsin, Wauwatosa, Wisconsin, United States; 2 Medical College of Wisconsin, Milwaukee, Wisconsin, United States; 3 Medical College of Wisconsin, Medical College of Wisconsin/Milwaukee, Wisconsin, United States

## Abstract

Despite the growing population of older adults, the geriatrics workforce has not similarly expanded. The number of geriatrics fellows has declined by 14.3% from 2012-2017. Implementation of innovative training programs may improve this reality. In 2002, the Medical College of Wisconsin (MCW) created the first four-year combined medicine residency and geriatrics fellowship (Med-Ger). Similar programs are currently being developed. The aim of this study is to describe the outcomes of the MCW Med-Ger program. Primary endpoints: American Board of Internal Medicine (ABIM) pass rates, ABIM Geriatric Medicine Certification pass rates, fellowship completion rates, and geriatric-focused practice. Results: There was a Med-Ger program fill rate of 73.7% (n=38). There was equivalent ABIM pass rate of 100% between Med-Ger graduates (n=18) and traditional graduates (n=25). Med-Ger trainees were more likely to complete their geriatrics fellowship (94.4% vs. 88%) and practice in geriatric-focused careers (82.4% vs. 68.2%). These outcomes suggest the benefit of a combined program for training future geriatricians. The MCW Med-Ger fill rate exceeds the national geriatrics fellowship fill rate of under 50%. Additionally, graduates may be more likely to practice geriatric medicine. This may help address population needs for an increased geriatrics workforce. In 2020, the ACGME approved an Advancing Innovation in Residency Education (AIRE) Medicine-Geriatrics Integrated Residency and Fellowship national pilot program. Further investigation of why trainees choose Med-Ger training and are more likely to continue with careers in geriatrics is needed in order to replicate the success of the MCW Med-Ger program.

